# Test-retest reliability of the single leg stance on a Lafayette stability platform

**DOI:** 10.1371/journal.pone.0280361

**Published:** 2023-01-17

**Authors:** Nureen Zaghlul, Siew Li Goh, Rizal Razman, Mahmoud Danaee, Chow Khuen Chan

**Affiliations:** 1 Department of Biomedical Engineering, Faculty of Engineering, Universiti Malaya, Kuala Lumpur, Malaysia; 2 SEMREG, Sports Medicine Unit, Faculty of Medicine, Universiti Malaya, Kuala Lumpur, Malaysia; 3 Centre for Sport & Exercise Sciences, Universiti Malaya, Kuala Lumpur, Malaysia; 4 Department of Social and Preventive Medicine, Faculty of Medicine, Universiti Malaya, Kuala Lumpur, Malaysia; Liverpool John Moores University, UNITED KINGDOM

## Abstract

The validity and reliability of the Lafayette stability platform are well-established for double leg testing. However, no evaluation of single leg (SL) stance on the platform was discovered yet. Therefore, this study aimed to investigate the reliability of conducting the SL stance on the Lafayette platform. Thirty-six healthy and active university students (age 23.2 ± 3.2 years; BMI 21.1 ± 3.1 kg/m^2^) were tested twice, one week apart (week 1; W1, week 2; W2). They stood on their dominant leg with eyes-open (EO) and eyes-closed (EC) in random order. Three successful trials of 20 seconds each were recorded. The duration during which the platform was maintained within 0° of tilt was referred to as time in balance (TIB). At all-time points, TIB was consistently longer in EO (EO_W1_: 17.02 ± 1.04s; EO_W2_: 17.32 ± 1.03s) compared to EC (EC_W1_: 11.55 ± 1.73s; EC_W2_: 13.08 ± 1.82s). A ±10 seconds difference was demonstrated in the Bland-Altman analysis in both EO and EC. Lower standard error of measurement (SEM) and coefficient of variation (CV) indicated consistent output. High intraclass correlation coefficient (ICC) values were seen between weeks (EO = 0.74; EC = 0.76) and within weeks (EO_W1_ = 0.79; EO_W2_ = 0.86; EC_W1_ = 0.71; EC_W2_ = 0.71). Although statistical measures (i.e., SEM, CV, and ICC) indicated good reliability of Lafayette for SL tasks, the wide agreement interval is yet to be clinically meaningful. Factors underlying the wide variation need to be identified before Lafayette is used for TIB assessment.

## Introduction

An acceptable degree of reliability is essential for the instruments used in clinical testing. Reliability is defined as the reproducibility of measurements [1]. In other words, reliability refers to the ability of a tool in producing consistent measurements over time during test-retest evaluation [2, 3]. Balancing apparatus with established reliability ranged from simple wobble board [4] to more sophisticated systems, such as force plates [5, 6], Biodex stability system [7, 8], and Computerized Dynamic Posturography (CDP) [9].

Lafayette stability platform is a system that utilizes an unstable base to assess dynamic balancing tasks, especially in double leg (DL) stance conditions [10–12]. Lafayette system is comparatively more versatile than some other existing systems because it allows the users to set their own test parameters, including testing time and angle limits to suit their needs. This system offers a good alternative when initiating assessment of subjects with impaired balance as it involves intuitive side-to-side uniplanar, and not multiplanar movements. Tilting of the platform from the horizontal plane against time can be captured by the Lafayette system [13]. A wide variation in the tilt angle is suggestive of impairment in postural control. Good postural control relies on the integrity of the somatosensory and neuromotor systems which include visual, vestibular, and ankle proprioceptive signals [14, 15].

Balance performance plays a vital role in maintaining daily functions and human maneuvering [14], especially among athletes [16]. A good sense of balance and lower limb strength [17] are essential in the execution of an immediate change of direction in a game [18]. Athletes who fail to fully maneuver their body control, speed, and sense of balance while changing direction commonly suffer lower extremity injuries such as ankle sprain [19]. Thus, for these individuals to be able to return to play rapidly, balance training is prioritized to improve the recovery rate, and balance tests are used to determine return to playtime [20].

The Lafayette stability platform has been utilized widely for balance training, particularly in the double leg (DL) stance condition. These studies namely investigated the effect of barefooted or in shod [21], the influence of dynamic training between genders [12] or the balance assessment using the saccadic eye movements on postural stability on unstable platforms [22]. All these research protocols implemented DL stance exclusively. Although the DL stance is undeniably important in human posture, a single leg (SL) stance is equally essential by taking into consideration that both DL and SL movements are used in daily life as well as in sports [23]. Balance performance on the Lafayette stability platform is usually measured as time in balance (TIB) i.e. the duration during which the platform is stabilized before it eventually deviates beyond a pre-determined angle.

Researchers frequently used the SL stance in assessing postural control, which includes improving postural control among Parkinson’s patients [24], enhancing the attentional focus in children with autism [25], and determining the risks of fall among elders [26], however, SL stance has not been performed on the Lafayette stability platform. Currently, no known study has published the reliability of the Lafayette stability platform for SL stance testing. On the other hand, for DL stance, Murray [27] was one of the first to demonstrate convincing results from the study of validity and reliability of the Lafayette stability platform. Murray [27] reported that thirty students volunteered for the motor balance testing using DL stance, with 0.91 product-moment correlation attained from the test-retest assessment. In addition, day-to-day evaluation on the technical part (i.e. horizontal maximum inclination of each side of the platform) showed a 0.002-microvolt difference. Hence, it was concluded that the Lafayette stability platform is a reliable instrument for motor balancing [28]. More recently, more studies also cited the platform as a reliable measure of balance for DL stance [28–30]. Considering its potential for use in a range of testing requirements and study population, it is important to determine its reliability. This study aimed to investigate the reliability of conducting the SL stance on the Lafayette stability platform. Lafayette stability platform may be an alternative for training, rehabilitation, as well as balance assessment apparatus regardless of the DL or SL stance tasks. It was hypothesized that the Lafayette stability platform will demonstrate acceptable agreement between weeks and satisfactory test-retest evaluation due to its simplicity and robust design.

## Methodology

### Participants

A total of thirty-six healthy and physically active university students (17 males, 19 females; age 23.2 ± 3.2 years; height 1.7 ± 0.1 m; weight 59.8 ± 11 kg; BMI 21.1 ± 3.1 kg/m^2^) were recruited between January 2021 and December 2021. The study was conducted in the Psychomotor laboratory, UM Arena. This study was approved by the Medical Research Ethics Committee (MREC) of the University of Malaya Medical Centre (201984–7710) and in accordance with the Declaration of Helsinki. The protocols and flow of the testing were explained to the participants prior to commencement. All participants provided a written consent form and were reimbursed for their participation.

An a priori sample size calculations were performed using G*Power statistical software, whereby a minimum of 19 participants were required to achieve an effect size of 0.80 and power of 90% [5]. Inclusion criteria were both male and female aged between 18 and 25 years old, physically active with no musculoskeletal pain that may affect the testing. Physically active was defined as having the regular exercise of at least 30 min per day of at least 3 days a week [21]. Participants with prior experience with the stability platform were excluded from the study to avoid any random bias during testing. In addition, participants with lower extremity injury (acute or overuse) that prevented them from participating in sports activities for at least one day in the previous 6 months were also exempted [21] to minimize the external factors that might affect the result.

### Lafayette stability platform setup

Lafayette Stability Platform Model 16030 (Lafayette, Indiana, USA) that was used consisted of a 65 x 107 cm solid wooden platform, allowing a maximum deviation of 15° from the horizontal to either side of the platform. The platform was placed 0.16 m from the frame and 0.22 m from the floor, a safety rail was mounted at the front to prevent participants from falling if they lose their balance [13].

Initially, the connection of the Universal Serial Bus (USB) cable of the platform with the Psymlab software in the computer was checked. A pre-test was conducted to verify the designation of the protocols (i.e. name of the task, number of trials, duration of each trial). The SL stance was set for the 20s of each trial in the Psymlab. A beep sound was produced at the start and end of testing for each trial. Additionally, the results of each participant were examined to ensure the tilting angle was recorded for each trial per participant.

### Protocol

Participants were required to do simple stretches and plyometrics of the lower limb [31] to prepare the muscles and avoid muscle cramps due to sudden movements, which include 10s of standing quadriceps and hamstring stretch, ankle plantar and dorsiflexion active stretches, ankle eversion and inversion movements, and ending with 20 double leg hops.

**Fig 1:**
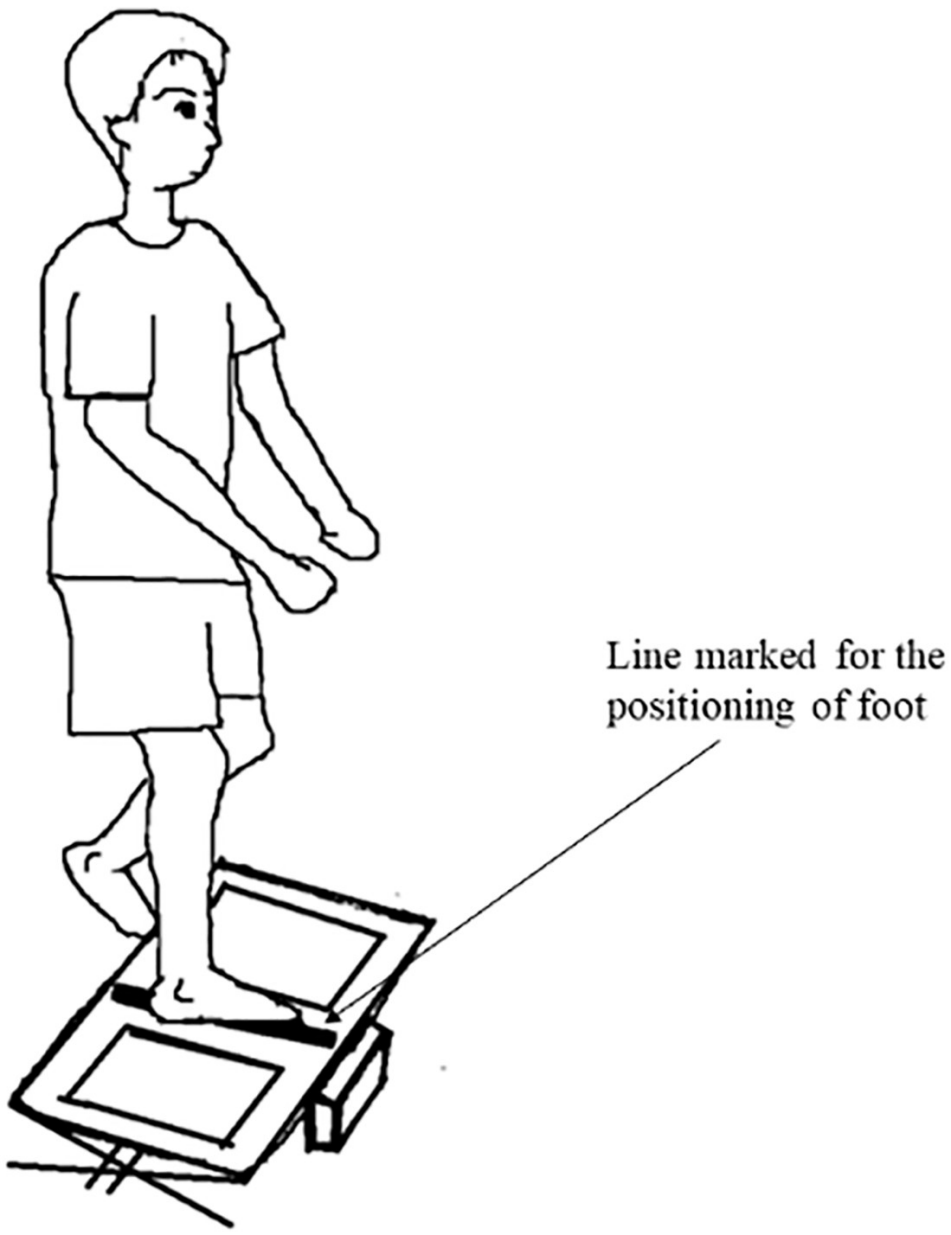
A participant performed a single leg (SL) stance in eyes-open (EO) condition. The dominant leg was positioned on the line marked in the middle of the platform.

Participants were tested only with their dominant leg with no assessment on the non-dominant leg, in which the dominant leg was defined as the leg used to kick a ball [32]. The ball kick test is a simple and reliable test in differentiating limb dominance [33]. The use of dominant leg in SL reliability is common because the lower limb balance was affected by the strength of the dominant leg [34]. Participants positioned their dominant leg on the line marked in the middle of the platform [35]. While barefooted, the participants stood on their dominant leg while the contralateral leg was lifted approximately 10 cm above the platform. The arms were allowed to hang at the side (Fig 1) [8]. The participants were required to balance under two testing conditions, eyes-open (EO) and eyes-closed (EC), in random order. The participants held the rail and were positioned in the SL stance. They were asked to release their hands and maintain balance once the test start (a beep sound was generated). Participants were allowed to make multiple attempts until they achieve three successful trials, whereby they are able to keep their balance for at least 20s [36]. The TIB measurements obtained from the three successful trials were averaged to reduce the variability error. The low number of trials was to negate the potential of learning effect and to avoid fatigue [8]. The trials were repeated if the non-dominant leg touched the platform or if the participants lost their balance before the 20s is up. The same protocol was applied a week later. Participants were advised to refrain from intense physical activity and to report any injuries during the week. The same tester performed the experiment in both weeks to avoid random bias in the experiment setting.

Using the Psymlab software (Lafayette Instrument, version 1.1.1.25998), time in balance (TIB) between the two testing sessions was recorded. TIB (in seconds) was defined as the duration in which the platform was maintained within the range of 0° from the horizontal plane. The longer TIB indicated better postural control.

### Statistical analysis

Comparison of the data from thirty-six participants between two different time points (i.e. Week 1, W1 and Week 2, W2) and between trials for each testing condition (i.e. EO and EC) were computed using MedCalc statistical software version 20.009 (Medcalc Software Ltd, Ostend, Belgium). Along with 95% limits of agreement (LoA), Bland-Altman analysis was performed to assess the agreement between data sets obtained at W1 and W2. A scatterplot was constructed, where the differences between two measurements (W1-W2) were plotted against the mean of two measurements ((W1+W2)/2). The 95% LoA is defined as ±1.96 standard deviation (SD) [37]. No significant difference between the measurements was reflected if the line of equality were within the interval [37]. A narrower 95% LoA indicated better agreement between measurements [38]. Additionally, intraclass correlation coefficient specifically model 3, k form (ICC3,k) for a two-way model, average measures, and absolute agreement were computed to quantify the reliability of the measurement [39]. To interpret the ICC values, the ICC classification of Barbado et al. [40] (less than 0.5, low; between 0.5 and 0.69, moderate; between 0.70 and 0.89, high; between 0.9 and 1.00, excellent) was applied. The level of significance was set at 0.05. Absolute reliability using standard error of measurement (SEM) (precision) was also determined using the following formula [8]. SD is the mean SD of W1 and W2 [8]. The measurements were considered reliable when SEM is less than 1 [41].


$$SEM=SD\sqrt{1-ICC}$$ (1)

Lastly, the reliability of the platform was further confirmed using the coefficient of variation (CV). Both intersubject (between-subject) and intrasubject (within-subject) variability were calculated to assess the reproducibility of the SL stance on the platform [42]. The reproducibility was considered acceptable when CV is <30% [43].

## Results

The test-retest reliability of the SL stance on a Lafayette stability platform was determined over two different sessions one week apart. The findings of the mean TIB calculated from three trials of the 20s each during EO and EC were summarized in Table 1.

**Table 1:** Lafayette stability platform test-retest reliability evaluation.

Mean time in balance (TIB) (s)	W1	W2	SEM	ICC
**Eyes-open (EO)**	17.02 ± 1.04	17.32 ± 1.03	0.53	0.74
**Eyes-closed (EC)**	11.55 ± 1.73	13.08 ± 1.82	0.87	0.76

Note. TIB: time in balance, s: second, W1: Week 1, W2: Week 2, SEM: standard error of measurement, ICC: intraclass correlation coefficient

**Fig 2:**
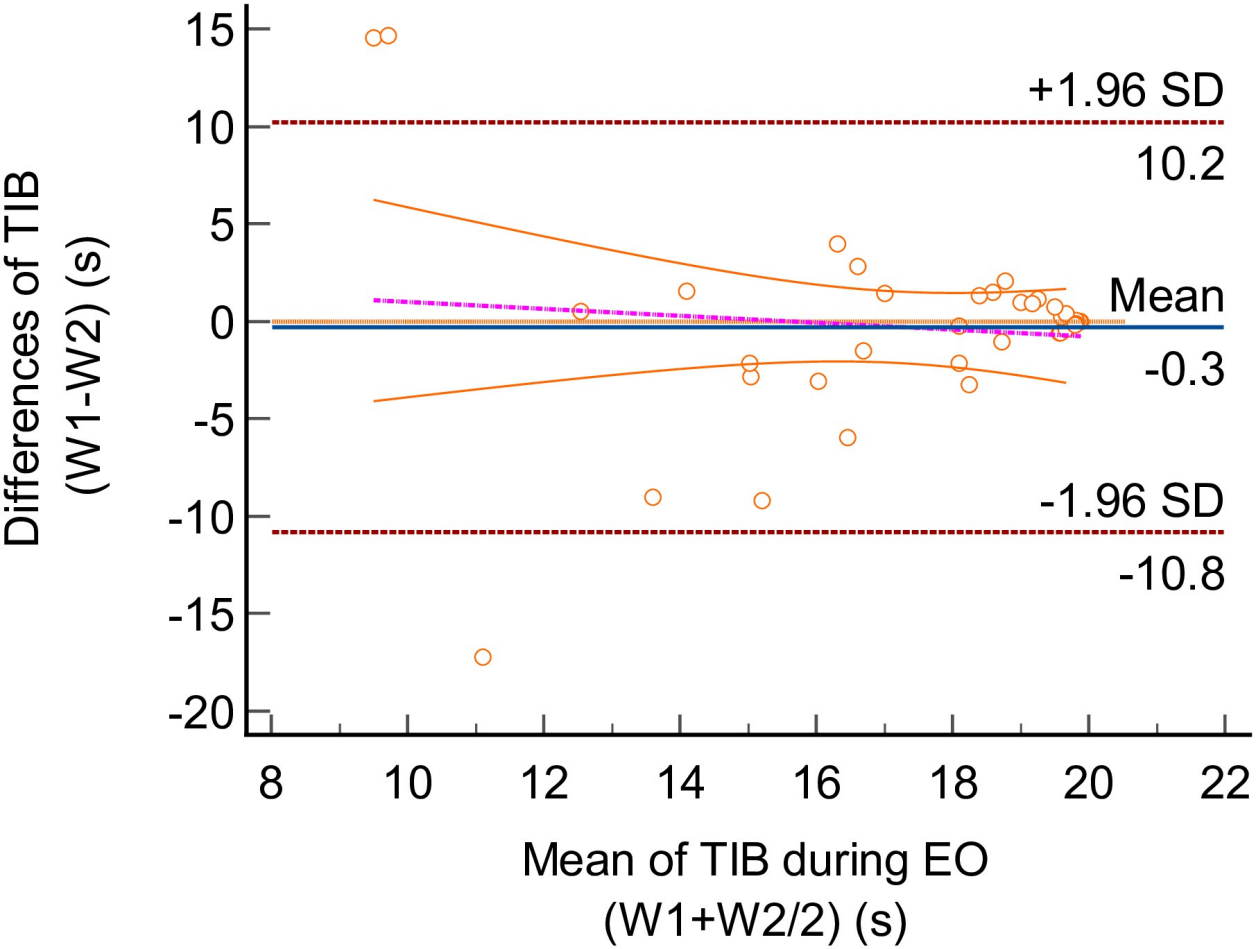
Bland-Altman plot for the time in balance (TIB) of the single leg (SL) stance task in eyes-open (EO) condition. The differences between Week 1 (W1) and Week 2 (W2) were plotted against the mean of W1 and W2. The solid line (blue) indicated the mean difference (bias). The interval between upper and lower limits represented the 95% Limit of Agreement (LoA).

**Fig 3:**
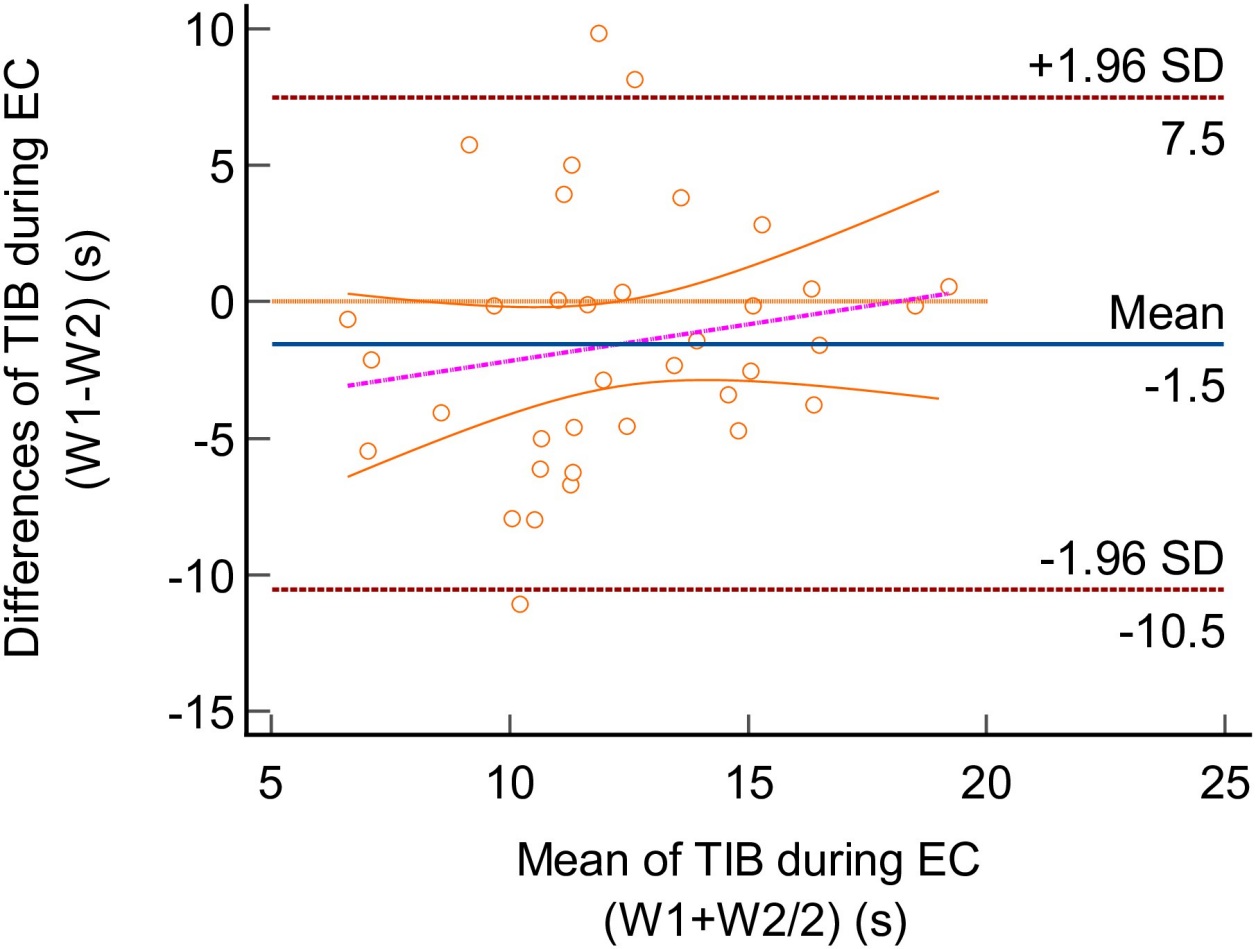
Bland-Altman plot for the time in balance (TIB) of the single leg (SL) stance task in eyes-closed (EC) condition. The differences between Week 1 (W1) and Week 2 (W2) were plotted against the mean of W1 and W2. The solid line (blue) indicated the mean difference (bias). The interval between upper and lower limits represented the 95% Limit of Agreement (LoA).

Using the Bland-Altman plot, the TIB differences between trials and LoA for the two testing conditions (i.e EO and EC) were illustrated in Figs 2 and 3. The mean difference (bias) between EO_W1_ and EO_W2_ was -0.30s (95% confidence interval (CI) -0.211 to 1.51). Upper and lower LoA for EO were 10.19s (95% CI 7.07 to 13.31) and -10.79s (95% CI -2.73 to -1.72). Whereas the EC_W1_ and EC_W2_ had a mean difference of -1.53s (95% CI -3.09 to 0.02). Upper and lower LoA for EC were 7.48s (95% CI 4.81 to 10.16) and -10.54s (95% CI -13.22 to -7.86).

Based on the Bland-Altman plot, the scatterplot graph demonstrated most of the TIB points were evenly distributed within the interval in both EO and EC. There were few outliers detected beyond the interval, however, the majority of the TIB points were within LoA. Furthermore, the lines of equality were within the LoA and close to the mean difference for both EO and EC. As such, this showed that no significant trend existed in the TIB data between the two weeks, which indicated the absence of systematic bias. Additionally, the *p*-value reported from the Bland-Altman analysis indicated no significant difference between the two data sets (W1 vs W2) in both conditions (EO, *p* = 0.736; EC, *p* = 0.054).

The reliability of the platform was further assessed using ICC. The computed ICC between weeks demonstrated high ICC values during EO (ICC = 0.74, 95% CI = from 0.58 to 0.85) and EC (ICC = 0.76, 95% CI = from 0.61 to 0.86) with appropriately moderate to high 95% CI between data sets. Moreover, the calculated ICC within the weeks (three consecutive trials) in each condition revealed high ICC values for all the trials (EO_W1_, ICC = 0.79; EO_W2_, ICC = 0.86; EC_W1_, ICC = 0.71; EC_W2_, ICC = 0.71).

As shown in Table 1, a lower SEM value during EO compared to EC condition indicated a more precise score in EO condition. Additionally, CV values for both inter- and intrasubject variability were below 30%, which is in the acceptable range. The intersubject variability ranging from 0% to 26.89% during EO and 1.21% to 28.49% during EC. While the intrasubject variability ranged from 0% to 26.61% during EO_W1_ and 0% to 27.17% during EO_W2_, whereas 1.05% to 27.78% during EC_W1_ and 1.37% to 29.2% during EC_W2_.

## Discussion

SL testing has been widely assessed on force plates and Biodex stability system, but usually not assessed using the Lafayette stability platform. Considering that SL testing are sensitive and able to distinguish postural impairment between injured and healthy individuals, thus, this study aimed to investigate the test-retest reliability of the SL stance on a Lafayette stability platform among physically active university students. Results indicate low SEM values, high ICC values, and lower subject variability, which suggest acceptable degree of reliability; however, the 95% LoA from the Bland-Altman plot was wide.

In this study, the reliability of the SL stance on a Lafayette stability platform was analyzed using a combination of reliability measures, unlike previous studies. Park et al. [44], in a systematic review of the evaluation of the test-retest reliability studies, found that the majority of the studies used only two types of statistical analyses in quantifying the instruments’ reliability. These included ICC and SEM [5, 8, 40], Bland-Altman and ICC [6], and ICC with correlation coefficient [45]. Hänninen et al. [46] applied an additional Wilcoxon ranked test to assess the mean difference between testing sessions. We, on the other hand, adopted a more rigorous method by using a combination of analyses (i.e. ICC, CV, SEM) including a graphical analysis of the Bland-Altman plot to enhance the confidence in our conclusion.

The Bland-Altman plot produced widely distributed data points of ±10s difference in the LoA, which may be considered a huge interval in the context of balance performance [47]. Compared to previous research, which proposed a difference of 7 points as a priori for the two measurements to achieve a clinical significance [48], LoA statistics in this study may not be representable, inferring a poor agreement based on the Bland-Altman analysis.

We had attempted to minimize the wide variation in the outcome by averaging the three trials, ensuring the measurements more similar to each other. This method is not uncommon and had been adopted by other researchers [49, 50]. As human performance can be inconsistent due to various factors, including the balance loss due to loss of concentration [36] and false foot positioning [35], using only one measure, either selecting the best reading or the first reading, may result in falsely wide variation in performance [51].

Despite the poor agreement in the Bland-Altman analysis, high ICC values in both conditions portrayed a reliable SL stance on the platform. No clear consensus has been reported on the applicable standard values for acceptable reliability using ICC. In an earlier study, Fleiss classified ICC generally as ≥0.75 to be excellent [52], however, the findings should be interpreted with caution in regard to the study’s field. Conversely, the ICC cutoff (i.e. threshold) adopted in this study was more relevant and has been used in previous reliability studies that assessed force platform’s postural sway measures [53] and gait using tri-axial accelerometer [54]. When compared with the other ICC values in the previous test-retest reliability studies of SL stance, Laessoe et al. [4] reported an ICC value of 0.87 on an instrumented wobble board, whereby a study of a dynamic SL stance using Biodex stability system quantified an ICC value of 0.65 [8] during EO condition. With high ICC values and appropriately moderate to high similarity of the 95% CI between data sets, the Lafayette stability platform is considered as a reliable tool for SL stance.

Lower SEM values in both conditions indicated higher precision of the TIB measurements between the trials [4, 8]. This implies the platform is able to produce consistent output at different time points. The evaluation of the reliability of the SL stance on a Lafayette stability platform was further strengthened with the coefficient of variation (CV). Findings in this study showed a CV of <30%, in inter-and intrasubject variability in both conditions, which were in the acceptable range for the field experiments. A similar CV threshold was reported in previous reliability studies, including the validation of devices [43, 55] to the fitness measurements [56], suggesting CV ranges of <30% were common in reliability studies. Hence, CV values in this study may be considered acceptable. Lower subject variability during EO suggested a homogeneity in the TIB measurements between weeks and repeated trials. These findings were common in the assessment with one type of population [42], in which this study engaged only physically active university students. Referring to Park et al. [44] and Atkinson and Nevill [51], we considered the statistical analyses performed in this study to be comprehensive and exhaustive.

Overall, the participants displayed better SL postural control on the platform during EO. This was demonstrated by the longer TIB measurements during SL stance and lower dispersion of TIB data points in the Bland-Altman analysis during EO compared to EC. It is believed that balance performance without vision is complicated and challenging. Barbierri et al. [57] denoted that insufficient visual information increases postural instability, which may reflect lower thresholds of postural responses in intermittent feedback control. Ku et al. [14] added that individuals rely more on vestibular and proprioception during EC, particularly on the ankle proprioception as the ankle-foot complex is the only part of the body contacting the ground [15]. In accordance with Ponce-González et al. [6], the tasks with EO yield more reliable results than with EC.

A SL stance on a Lafayette stability platform may stimulate ankle joint movement and instigate external perturbation if the user fails to control their balance. This may represent a challenging task, as the platform is unstable and deviates in the mediolateral direction freely. Compared to the other SL task such as Timed Unipedal Stance Test and Balance Error Scoring System, which uses manual timing and scoring [5], tilt measurement from the Lafayette stability platform could be more relevant and accurate [4]. Therefore, we would like to advocate the use of Lafayette stability platform as an alternative balance device. However, further investigations are still needed to elucidate the underlying factors (e.g. lower limb strength, perceived stability/confidence level) that are responsible for the wide variation in the results found here.

The present study had several limitations. The findings of this study may not be generalized to the general population as data were collected from the physically active students. Future studies may recruit larger cohorts in order to perform subgroup analysis to identify confounding factors for the wide LoA. Confounding factors can also be explored by recruiting other target populations (i.e. middle age/geriatric, those who had undergone rehabilitation and spinal cord injured survivors). Furthermore, the participants were tested barefoot in our study. Testing with and without shoes on the Lafayette stability platform may yield contradicting findings. Lastly, it may also be worthwhile to investigate if the reliability of the Lafayette platform will be compromised when fatigue sets in such as when more repetitions or trials were performed, or when subjects are required to maintain balance for longer period.

## Conclusions

The test-retest evaluation of the SL stance on the Lafayette stability platform showed acceptable agreement as measured by ICC, SEM, and CV in both EO and EC conditions except for LoA. A more rigorous recruitment criteria, in addition to being uninjured and physically active, may yield a better test-retest reliability score for the Lafayette stability platform for SL tasks.

## Supporting information

S1 File Bland-Altman data. (DOCX)

S2 File Standard error of measurement (SEM) and coefficient of variation (CV) data. (DOCX)
