# Supplementary material for: Test-retest reliability of the single leg stance on a Lafayette stability platform
Source: PLoS One. 2023 Jan 17;18(1):e0280361. doi: 10.1371/journal.pone.0280361 (PMC9844846; doi:10.1371/journal.pone.0280361)
Supplement: S1 File — (DOCX) [file pone.0280361.s001.docx]

# Bland-Altman plot

| Method A | meanEOW1 |
| --- | --- |
| Method B | meanEOW2 |

| Sample size | 36 |
| --- | --- |

| Option | Plot differences |
| --- | --- |

| Arithmetic mean | -0.3028 |
| --- | --- |
| 95% Confidence interval | -2.1140 to 1.5085 |
| P (H_0_: Mean=0) | 0.7364 |
| Lower limit | -10.7949 |
| 95% Confidence interval | -13.9194 to -7.6705 |
| Upper limit | 10.1894 |
| 95% Confidence interval | 7.0650 to 13.3138 |

| Regression Equation | y = 2.7579  +  -0.1782  x |
| --- | --- |

| Parameter | Coefficient | SE | t | P | 95% CI |
| --- | --- | --- | --- | --- | --- |
| Intercept | 2.7579 | 5.3917 | 0.5115 | 0.6123 | -8.1993 to 13.7151 |
| Slope | -0.1782 | 0.3096 | -0.5758 | 0.5686 | -0.8074 to 0.4509 |

# Bland-Altman plot

| Method A | meanECW1 |
| --- | --- |
| Method B | meanECW2 |

| Sample size | 36 |
| --- | --- |

| Option | Plot differences |
| --- | --- |

| Arithmetic mean | -1.5308 |
| --- | --- |
| 95% Confidence interval | -3.0863 to 0.02464 |
| P (H_0_: Mean=0) | 0.0535 |
| Lower limit | -10.5413 |
| 95% Confidence interval | -13.2246 to -7.8581 |
| Upper limit | 7.4797 |
| 95% Confidence interval | 4.7965 to 10.1629 |

| Regression Equation | y = -4.8199  +  0.2671  x |
| --- | --- |

| Parameter | Coefficient | SE | t | P | 95% CI |
| --- | --- | --- | --- | --- | --- |
| Intercept | -4.8199 | 3.2407 | -1.4873 | 0.1461 | -11.4057 to 1.7659 |
| Slope | 0.2671 | 0.2557 | 1.0445 | 0.3036 | -0.2526 to 0.7868 |
