# Supplementary material for: Test-retest reliability of the single leg stance on a Lafayette stability platform
Source: PLoS One. 2023 Jan 17;18(1):e0280361. doi: 10.1371/journal.pone.0280361 (PMC9844846; doi:10.1371/journal.pone.0280361)
Supplement: Supplementary file 2 [file pone.0280361.s002.docx]

n=36

ICC

SEM= SD√1−

√T [illegible]

	mean	sd	ICC	SEM	
EOW1	17.02	1.04			
EOW2	17.32	1.03			
meanEO	17.17	1.04	0.74	0.53	1.04(√1-0.74)
ECW1	11.55	1.73			
ECW2	13.08	1.82			
meanEC	12.31	1.78	0.76	0.87	1.78(√1-0.76)

CV= (sd/mean)*100

	range indi.	Cvintra
EOW1	0% - 26.61%	6.94
EOW2	0% - 27.17%	7.04
ECW1	1.05% - 27.78%	15.88
ECW2	1.37% - 29.2%	15.14

Cvinter	
EO	6.99
EC	15.51
